# The Changing Landscape of Akabane Virus: A Comprehensive Review

**DOI:** 10.3390/v18070770

**Published:** 2026-07-13

**Authors:** Celia Alonso, Iván Mazuecos-Aragonés, Javier Ortego, Gema Lorenzo, Luis Jiménez-Cabello

**Affiliations:** 1 Centro de Investigación en Sanidad Animal, Instituto Nacional de Investigación y Tecnología Agraria y Alimentaria, Consejo Superior de Investigaciones Científicas (CISA-INIA, CSIC), Valdeolmos, 28130 Madrid, Spain; celia.alonso@inia.csic.es (C.A.); ivan.mazuecos@inia.csic.es (I.M.-A.); ortego@inia.csic.es (J.O.); lorenzo.gema@inia.csic.es (G.L.); 2 Escuela de Doctorado, Universidad Autónoma de Madrid (UAM), 28049 Madrid, Spain

**Keywords:** Akabane virus, orthobunyavirus, ruminants, Culicoides vector, transplacental transmission, arbovirus

## Abstract

Akabane virus (AKAV) is an orthobunyavirus transmitted by biting midges of the genus *Culicoides*. This virus mainly affects ruminant species and represents an important threat to animal health across the world, being distributed in regions of Asia, the Middle East, Africa, and Oceania. Although adult animals usually develop subclinical infection, AKAV can cross the placental barrier producing abortions, stillbirths, and severe congenital malformations that lead to substantial economic losses. Lately, AKAV has expanded its host range to non-ruminant species such as domestic pigs and bamboo rats, showing a marked virulence in these mammalian species. The host plasticity of AKAV together with the expanding distribution of Culicoides biting midges induced by global warming raise concerns about the potential arrival of AKAV into previously unaffected regions with naïve livestock populations. Due to the absence of therapeutic treatments, vaccination remains the main measure against AKAV. As climate change promotes vector expansion, AKAV can be considered as an increasingly relevant emerging pathogen in animal health, which enhances the need for strengthened surveillance programs, a deeper understanding of the virus biology, virulence and pathogenicity, and the development of novel vaccine candidates against this viral pathogen. This review provides an overview of current knowledge about AKAV biology, epidemiology, and disease, and a discussion about control strategies.

## 1. Introduction

Arthropod-borne viruses represent a continuous global challenge to human and animal health due to the expansion of competent vectors to non-endemic regions motivated by climatic, ecological, and anthropogenic factors. Regarding animal health, vulnerability of livestock populations to arboviruses has been evident in recent years, with the striking example of the first incursion and wide distribution of Epizootic hemorrhagic disease virus (EHDV) in several European countries [1,2]. Among these pathogens, Akabane virus (AKAV) remains a major concern for ruminant production systems. AKAV belongs to the genus *Orthobunyavirus* within the family *Peribunyaviridae* (order Ellioavirales) [3]. Traditionally, AKAV is a member of the Simbu serogroup along with other relevant viruses in animal health such as Schmallenberg virus (SBV), Aino virus (AINOV) or Shamonda virus (SHAV), among others [4,5,6]. Orthobunyaviruses belonging to the Simbu serogroup are frequently associated with reproductive disease and congenital malformations in livestock, being transmitted primarily by Culicoides biting midges [4]. Since its first identification in Japan in 1959 and the major Japanese outbreaks between 1972 and 1974 where more than 42,000 abnormal calves were born [7,8], AKAV has caused recurrent epizootics across Asia, the Middle East, Africa, and Oceania, where it is responsible for significant reproductive losses, including abortions, stillbirths, and severe congenital malformations.

Significant knowledge gaps remain regarding AKAV. Control of AKAV relies exclusively on vaccination with classical vaccine approaches. Nonetheless, antigenic variability among AKAV strains, genomic reassortment events and safety concerns associated with live-attenuated vaccines (LAVs) highlight the need for next-generation vaccine candidates against the virus and the importance of surveillance programs. In this review, we synthesize current knowledge on AKAV biology, pathogenesis, epidemiology, host range, and the immune response against the virus, and discuss challenges and perspectives for mitigating the impact of this increasingly relevant arboviral pathogen.

## 2. Akabane Virus

Viruses of the genus *Orthobunyavirus* are spherical particles of approximately 100 nm in diameter characterized by a lipidic envelope that contains a segmented negative sense single-strand RNA genome ((−)ssRNA) flanked by 3′ and 5′ UTRs [9,10]. The segmented genome consists of three segments designated as S (small), M (medium), and L (large) that encode four structural proteins and two additional non-structural proteins [11,12].

**Figure 1:**
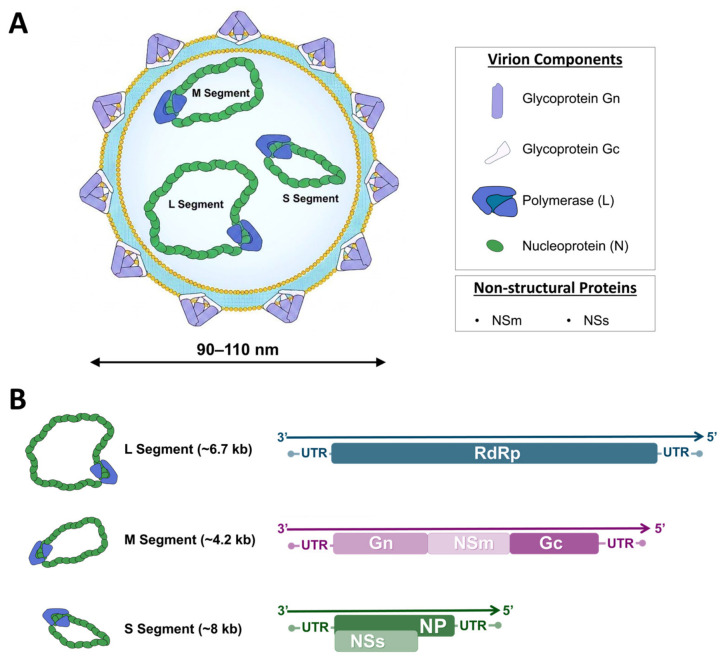
(**A**) Diagrammatic representation of the viral particle of AKAV. The AKAV particle consists of an enveloped, spherical virion containing a tri-segmented, single-stranded, negative-sense RNA genome (S, M, L segments) in association with the N protein and the viral RdRp, forming circular ribonucleoprotein complexes. Glycoproteins Gn and Gc protrude outwards from the viral envelope. Two additional non-structural proteins (NSm and NSs) are expressed during the replicative cycle. (**B**) Schematic representation of the genomic structure of AKAV. The L segment is monocystronic whereas the M and S segments are polycistronic. The M segment encodes a polyprotein that is cleaved into proteins Gc, Gn and NSm. The S segment contains an ORF that encodes the N protein and an overlapping ORF encoding the NSs protein.

**Figure 2:**
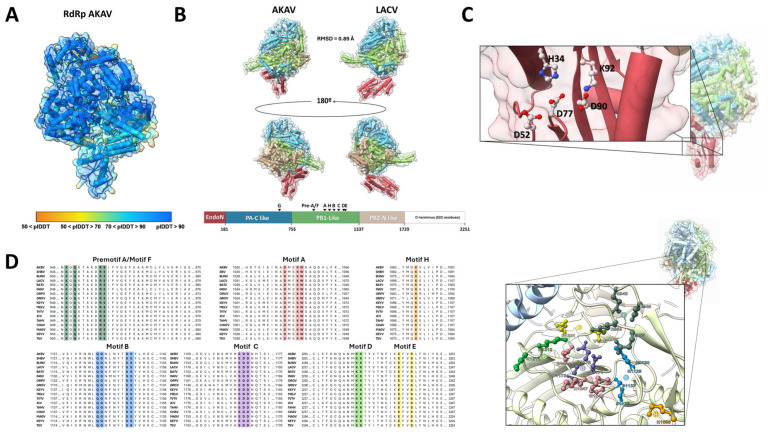
Structural organization of the RdRp of AKAV. (**A**) Predicted tertiary structure of the AKAV RdRp. Protein prediction was performed using AlphaFold2 (GenBank accession number: BAF57206). Colored regions represent the local confidence in the structure (pLDDT score; blue—very high, light blue—high, yellow—low, orange—very low). (**B**) Predicted structure of AKAV RdRp and X-ray crystal structure of LACV RdRp protein (PDB: 5AMQ). Colored regions represent the conserved functional domains of the RdRp: N-terminal Endonuclease domain (Endo N; red), PA-C-like domain (blue), PB1-like domain (green), and PB2-N-like domain (beige). (**C**) Detailed view of the N-terminal Endonuclease domain of AKAV RdRp. The active site of the Endonuclease domain (residues 1–181; GenBank accession number: AB190458) within the AKAV RdRp structure prediction is shown, with key residues in the active site indicated. (**D**) Multiple sequence alignment (MSA) of the PB1-like catalytic core of the orthobunyavirus RdRp. Conserved residues from premotif A/F to motif E are shown in bold and highlighted in shades of the corresponding motif colors: motif F in greenish blue; motif A in pink; motif F in orange; motif C in purple; motif B in green and motif E in yellow. The corresponding residues are mapped onto the RdRp catalytic site in the structural representation. Virus abbreviations and GenBank accession numbers are as follows: Schmallenberg virus (SBV), AGU16235; Bunyamwera virus (BUNV), CAA32553; La Crosse virus (LACV), ADE28877; Batai Virus (BATV), ALN13241; Ingwavuma virus (INGV), AHY22331; Oropouche virus (OROV), AAO11841; Guaroa virus (GROV), AKC42494; Keystone virus (KEYV), ALD52506; Melao virus (MELV), APA28993; Trivittatus virus (TVTV), APA34119; Jamestown Canyon virus (JCV), QCF29631; Tahyna virus (TAHV), AGZ03703; Chatanga virus (CHATV), AHI87734; Maguari virus (MAGV), ARI46638; Keystone virus (KEYV), ALD52506; Tensaw virus (TSV), AXP32066. The structural model shown corresponds to an AlphaFold2 prediction and should be considered as a hypothetical representation pending experimental validation.

The L segment contains a single Open Reading Frame (ORF) that encodes the viral RNA-dependent RNA polymerase (RdRp) (~260 kDa) essential for genome replication and transcription of viral genome (Figure 1B) [13]. For AKAV, the RdRp has been proposed as a potential virulence determinant [14] as temperature-sensitive RdRp mutants correlated with attenuation in vivo. Although no structural studies of the AKAV RdRp are currently available, the high structural conservation of viral RNA polymerases within the family *Bunyaviridae* [15] allows for its study through comparative structural modeling. Thus, the structural prediction of the AKAV RdRp showed high similarity (RMSD of 0.89 Å) to the crystal structure of the RdRp of La Crosse virus (LACV; PDB: 5AMQ) [16] (Figure 2B). The Influenza virus RdRp served as a reference for the structural and functional characterization of the bunyaviral RdRp, such as those of LACV (genus *Orthobunyavirus*) [16], Rift Valley fever virus (RVFV; genus *Phlebovirus*) [17], Crimean-Congo hemorrhagic fever virus (CCHFV; genus *Orthonairovirus*) [18], severe fevers with thrombocytopenia syndrome virus (SFTSV; genus *Bandavirus*) [19] or Tomato Spotted Wilt Virus (TSWV; genus *Tospovirus*) [20], identifying four main functionally equivalent regions [15,16,21,22]: the N-terminal endonuclease domain (aa 1–181), the PA-C-like domain (aa 182–755), the PB1-like domain (aa 756–1337), and the PB2-N-like domain (aa 1338–1729) (Figure 2B). The N-terminal endonuclease domain, conserved across the *Bunyaviridae* family, is responsible for cleaving capped host mRNAs [15,21,23,24]. This endonuclease activity represents a cap-snatching mechanism that mainly serves to provide capped oligonucleotides that prime viral mRNA transcription, resulting in capped but usually non-polyadenylated viral transcripts [11]. Sequence-based analyses support the presence of an N-terminal endonuclease domain in the AKAV RdRp. This is consistent with the highly conserved bunyaviral endonuclease signature motif (H…P D…D/E…K) reported at the N-terminus of diverse bunyaviral RdRp, with AKAV exhibiting conserved active site residues (H34, D52, D77, D90, K92 and K106) [15,21] (Figure 2C), which suggests that AKAV also exploits cap-snatching activity to initiate viral mRNA synthesis. Protein sequence comparison between AKAV and LACV RdRps allowed for the identification of the PA-C-like domain (Figure 2B), which is equivalent to the C-terminal PA domain of Influenza virus RdRp [16]. Conserved polymerase motifs are structurally and functionally relevant elements of the RdRp. The canonical motifs A–D were first described [25,26]. Subsequently, a total of eight conserved motifs (A–H) were recognized, defining the catalytic core and adjacent functional region [16,27]. Motif G has been described within the PA-C-like domain and contains a conserved arginine residue that has been proposed to participate in the positioning of the initiating nucleoside triphosphate (NTP) at the active site [16,18,28]. The PB1-like domain adopts the classical right-hand fold (finger-palm-thumb) subdomain arrangement that contains the catalytic core responsible for RNA synthesis (Figure 2B) [16]. Catalytic motifs A–H, located in the palm subdomain and highly conserved within the *Bunyaviridae* family, coordinate template RNA binding, nucleotide binding, and catalytic cofactor coordination [16] (Figure 2D). Finally, the PB2-like domain acts as a cap-binding domain as it plays a key role involved in recognition and binding of the RdRp to the cap structure of host mRNAs, an essential step for the proper initiation of viral RNA transcription [15,23]. As our AKAV RdRp model is based on an AlphaFold2 hypothetical structural prediction, structural and mutational analyses are still needed to elucidate the structural and functional properties of the AKAV L protein domains and to determine singularities of the AKAV RdRp to fully understand the molecular determinants of AKAV replication efficiency and virulence.

The M segment of AKAV encodes a polyprotein that is cleaved by host proteases into the transmembrane glycoproteins Gc (~100–125 kDa) and Gn (~35–40 kDa), and the non-structural protein NSm (Figure 1B) [12,29]. In the mature virion, Gn and Gc form heterodimers that assemble into tripod-like spikes composed of three Gn–Gc heterodimers on the lipidic envelope. This structural organization is conserved among orthobunyaviruses, although some variability in surface arrangement has been described [10,30,31,32]. Despite the fact that several receptors and cofactors are involved in bunyavirus attachment and entry [33], the specific host factors involved in AKAV cell attachment and entry remain poorly characterized. Nevertheless, in vitro studies have shown that heparan sulfate proteoglycans (HSPGs) act as important cellular attachment factors for AKAV entry [34]. After binding to the cell surface, AKAV enters mammalian cells through alternative endocytic pathways such as clathrin-mediated or dynamin-dependent endocytosis pathways. In both cases, viral penetration requires endosomal acidification that leads to a low pH-triggered membrane fusion step [35]. The structural and functional characteristics of the AKAV Gc protein have been extensively revised elsewhere [34,36]. Structural studies have revealed that orthobunyavirus Gc protein displays a modular structure, consisting of a highly variable N-terminal half that includes the exposed head domain, and a more conserved C-terminal region functionally associated with membrane fusion [37,38]. As it is a key determinant for cell receptor recognition, the Gc protein, specially the head domain, is the main target of virus neutralizing antibodies (nAbs) [38,39], which increases sequence variability among AKAV isolates due to immunological pressure. Early studies based on neutralizing monoclonal antibodies defined at least five antigenic regions in the AKAV Gc protein [40]. Subsequent studies enabled the experimental mapping of two domains containing neutralizing epitope and spanning residues 1–97 and 189–397 [41], as well as a broadly neutralizing linear epitope located at residues 1134–1142 [42], suggesting that the antigenicity of AKAV Gc protein is distributed across functionally distinct regions of the protein. In addition to cell attachment, a recent work has described the potential association of the AKAV Gc glycoprotein with cellular factors such as FKBP8, BNIP3 and USP30 mitochondrial proteins [43], which play roles in protein trafficking and folding, mitochondrial homeostasis, autophagy and mitophagy [44,45,46]. These cellular proteins alter virus replication, but the exact mechanism has not been studied so far.

The transmembrane Gn glycoprotein, relatively well conserved across orthobunyaviruses, plays essential roles in Gc–Gn spike architecture, intracellular trafficking and virion assembly [38]. Orthobunyavirus Gn protein stabilizes the tripod-like spike structure by its membrane-proximal position, partially shielding the Gc fusion loops [32], and acts as a chaperone for the correct trafficking of Gc to the Golgi complex where virion maturation occurs [12,32,38,47,48,49,50]. No structural or functional studies of the AKAV Gn protein exist. Although no published structures of the orthobunyavirus Gn protein are available, it has been identified that the bunyaviral Gn protein contains an ectodomain harboring N-linked glycans, a single transmembrane domain, and a short cytoplasmic tail that are involved in Golgi targeting and retention signal and interaction with the viral ribonucleoprotein (RNP) complex during assembly [48,51,52]. N-glycosilations are critical for proper protein folding, Gn–Gc heterodimerization and intracellular trafficking of both glycoproteins [53]. The specific structural and functional features of AKAV Gn remain insufficiently characterized and require further investigation.

The role of the NSm protein (~25–30 kDa), encoded by the polycistronic M segment [29] (Figure 1B), remains poorly characterized for AKAV and other bunyaviruses. NSm is present as an insertion between the two glycoproteins within the M polyprotein, being subsequently cleaved by cellular proteases during polyprotein maturation [54]. In the case of RVFV, the NSm protein is dispensable for virus replication in vitro although it exhibits an antiapoptotic role in infected cells [55]. In orthobunyaviruses such as SBV or BUNV, the NSm is also dispensable for virus replication in vitro [56,57,58,59,60] but it is involved in virus morphogenesis [61]. The role of NSm protein of AKAV is still unknown. Nonetheless, it is important for efficient virus replication in mammalian cells and may act as a virulence factor in vivo [62]. As recently seen for BUNV, the NSm protein also plays a prominent role as a crucial factor for virus dissemination in insect vectors by allowing for crossing the midgut barrier [63]. In contrast, such role has not been described for the NSm protein of SBV, as infection of Culicoides insect vectors was not influenced by impairment of NSm expression [64]. This may indicate that this vector species represents a more permissive organism for viral dissemination in which SBV NSm-dependent functions are not essential or can be compensated by alternative viral or cellular factors. Despite the fact that this could apply for AKAV, the potential role of the NSm protein of AKAV related to vector competence and dissemination should be further explored. These divergent phenotypes suggest that NSm may have virus-specific roles despite being structurally conserved, raising the possibility that AKAV NSm could participate in host- or vector-specific interactions yet to be elucidated.

**Figure 3:**
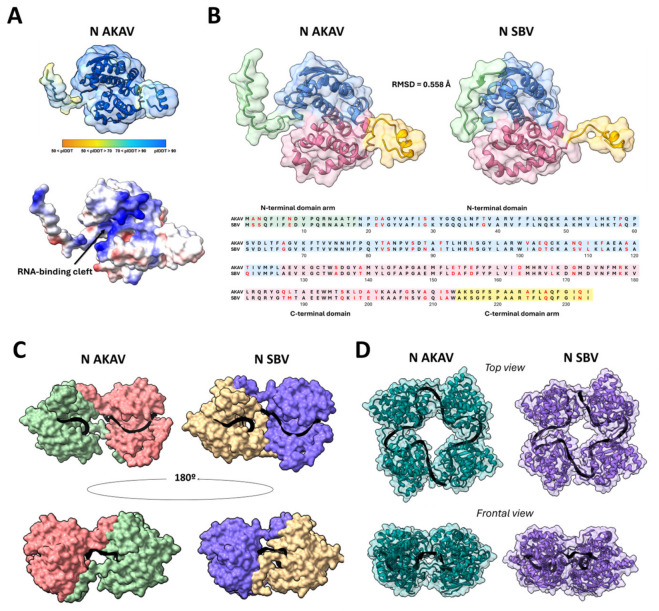
Structural organization of the N protein of AKAV. (**A**) Predicted tertiary structure of the AKAV N protein. Protein prediction was performed using AlphaFold2 (GenBank accession number: BAA24201). Colored regions in the upper model represent the local confidence in the structure (pLDDT score; blue—very high, light blue—high, yellow—low, orange—very low). The electrostatic surface of the AKAV N protein is shown in the lower model. The positively charged RNA-binding cleft is indicated. (**B**) Tertiary protein structures of the AKAV and SBV nucleoproteins (PDB: 4JNG). The N-terminal arm (green), the N-terminal domain (blue), the C-terminal domain (pink), and the C-terminal arm (yellow) are represented. N- and C-terminal flexible arms mediate monomers interaction. Protein sequence alignment is shown. Red letters indicate residue differences. (**C**) Dimeric assemblies of the AKAV and SBV nucleoprotein. AKAV monomers are shown in green and pink, and SBV monomers in yellow and purple. The RNA is represented in black. (**D**) Tetrameric assemblies of the AKAV (blue) and SBV (purple) nucleoproteins shown from the top and front view. The RNA is represented in black. The structural model shown corresponds to an AlphaFold2 prediction and should be considered as a hypothetical representation pending experimental validation.

The S segment, highly conserved among AKAV isolates [65,66], encodes the nucleocapsid (N) protein and the non-structural protein NSs in overlapping reading frames [67] (Figure 1B). The N protein (~19–26 kDa [68]) associates with each (−)ssRNA segment to form the RNPs in association with the viral RdRp [22]. The primary function of the N protein of AKAV (extensively reviewed in [68]) is the protection of the viral genome, with the RNPs acting as the functional replication and transcription units, being the active templates for the viral RdRp [15]. Sequence alignment of the AKAV N protein with its SBV homologs revealed a high degree of conservation as previously observed [69] (Figure 3B). Structurally, the crystal structure of the SBV N protein (PDB: 4JNG) [70] and the predicted hypothetical model of the AKAV N protein also showed robust similarity (RMSD of 0.558 Å), suggesting structural conservation and supporting the use of the SBV N protein as a model for comparative study of the AKAV N protein. Genome encapsidation depends on the interaction of the viral RNA with multiple copies of the N protein, which sequestered the RNA within its positively charged RNA-binding groove (Figure 3A) [71]. This process requires homotypic interactions between adjacent N monomers to form tetramers, consistent with the structural arrangement described for the N protein of SBV (Figure 3C,D) [72,73,74]. In addition, the N protein is also involved in the regulation of viral genome transcription, modulation of the viral RdRp activity and interaction with the Gc–Gn complex [9,12,74,75,76]. In line with other bunyaviruses, the AKAV NSs protein (~10 kDa as theoretically estimated using ProtParam, https://web.expasy.org/protparam/ (accessed on 10 July 2026), encoded by an overlapping ORF in the S segment [67], has been described as a virulence factor that contributes to viral distribution and disease severity in mammalian host [77,78,79]. The bunyavirus NSs protein develops multiple roles (extensively reviewed in [80]) including IFN antagonism [81], disruption of host cell transcription machinery [82,83] and promotion of autophagy [84], among others [80]. For AKAV, mutant deletion viruses lacking NSs expression show impaired viral replication and abolished fetal neuroinvasiveness in infected suckling mice and pregnant goats, pointing out its role as a key virulence factor [85,86,87]. The AKAV S segment also represents a suitable region for stable insertion of foreign sequences without compromising N or NSs expression. In this sense, recombinant AKAV harboring fluorescent or luminescent reporter genes in the S segment has allowed for the study of virus tropism or the detection of AKAV in biological samples, while maintaining virological and pathogenic features comparable to wild-type virus [88,89,90].

## 3. Changes in AKAV Epidemiology: Influence of Global Warming

**Figure 4:**
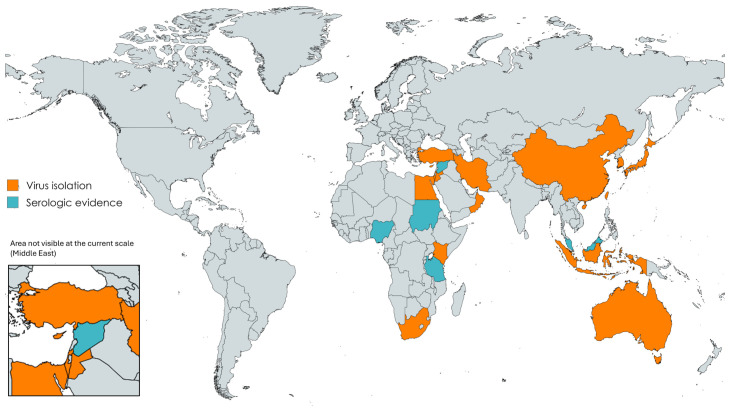
Distribution of AKAV across the world. AKAV is historically endemic to many countries of Asia, Africa, the Middle East and Oceania. Colored areas represent the countries where AKAV has caused outbreaks over the years (orange), or serological evidence of AKAV circulation exists (blue). Countries where outbreaks or serologic evidence are located within a specific region are indicated as infected in their entirety. The Mediterranean sector of the Middle East has been magnified to allow for clearer distinction among adjacent countries.

AKAV was first identified and isolated in Japan in 1959 [8], being recognized as the etiological agent of important outbreaks characterized by high rates of abortion, stillbirth and congenital malformations in cattle populations in subsequent years [7,91]. To date, this virus has been isolated in Asia, the Middle East, Africa, and Australia, regions where recurrent outbreaks occur [92,93,94,95]. Specifically, AKAV has been isolated in Japan [92,93,96,97,98], China [99,100,101,102,103,104,105], Taiwan [106,107], Republic of Korea [108,109], Oman [110], Iran [111,112,113], South Africa [114], Zimbabwe [115], Indonesia [116], Australia [117,118,119,120], and Kenya [121], and serological evidence also indicates the presence of AKAV in Sudan [122], Nigeria [123], Tanzania [124], Syria [125] and Malaysia [126] (Table 1) (Figure 4). Vector surveillance is critical to uncover hidden transmission cycles or predict the emergence and spread of AKAV and other arboviruses. Accordingly, although vertebrate infection has not been documented in several tropical and subtropical Asian countries, entomological surveys in neighboring regions suggest the potential circulation of AKAV in these Asian regions [127]. Interestingly, the Mediterranean Basin also represents a favorable environment for AKAV as it is present in Turkey [128,129,130,131], Cyprus [125], Jordan [125], Egypt [132,133] and Israel [134,135] (Figure 4). Phylogenetic studies have clustered the current AKAV isolates into four genogroups named I–IV. East Asian AKAV isolates were classified into Genogroups I and II, whereas Australian and African isolates were classified into Genogroups III and IV [65,66,136].

East Asia is one of the regions with the highest population density of domestic ruminants worldwide, becoming an ideal habitat for arboviruses affecting livestock [137,138]. Economic losses caused by AKAV epizootics are considered significant and are related to reduced fertility, stillbirth, abortion, congenital malformation, veterinary costs and decreased milk production [139,140]. In this context, Japan emerged as a hotspot for ruminant arboviral infections, which have severely impacted the livestock industry [94,138]. Among them, AKAV still constitutes a major factor that limits Japanese livestock productivity. In this country, AKAV Genogroups I and II have circulated, with cattle as the most affected host species. During Japanese epizootics events involving AKAV between 1972 and 1974, the predominant clinical manifestation of disease included abortions, stillbirths and congenital malformations in cattle manifested as an arthrogryposis-hydranencephaly syndrome [7]. Usually, AKAV infection of postnatal animals is subclinical, but it can lead to clinical signs such as astasia, dysstasia, ataxia and opisthotonus, and inflammatory lesions of the central nervous system such as encephalomyelitis [141]. AKAV isolates belonging to Genogroup I are mainly related with increased neurovirulence in postnatal cattle [92], whereas Genogroup II isolates are able to cause similar symptoms in cattle though these are extremely rare and are preferentially associated with classical arthrogryposis-hydranencephaly syndromes [92,142]. Since 2006, AKAV isolates of Genogroup I had a prominent role during Japanese outbreaks, which were characterized by severe encephalomyelitis in postnatal cattle [97,98], reflecting a shift in circulating genogroups and disease outcome [92]. In Republic of Korea, comparable severe epizootics of postnatal bovine encephalomyelitis caused by AKAV isolates of Genogroup I have been described [108]. Considering that the viruses causing encephalomyelitis outbreaks in Japan and Republic of Korea were closely genetically related, Yanase T. et al. (2019) hypothesized that the epidemiological origin of neurovirulent isolates (Genogroup I) of AKAV affecting postnatal cattle in Japan could be located in Republic of Korea [92]. This phenotypic shift in Japan towards a marked neurotropism/neurovirulence in postnatal animals exemplifies how geographical spread and genetic variability of AKAV can drive significant changes in viral pathogenicity [92], highlighting the importance of active cross-border transmission routes.

**Table 1:** First documented AKAV detections across regions.

Country	Year (First Detection/Isolation)	Genogroup	Host Source	Ref.
Japan	1959	Genogroup I * (JaGAr39 strain)	*Culex tritaeniorhynchus*	[8]
Australia	1968	Genogroup III (B8935)	*Culicoides brevitarsis*	[140]
Republic of Korea	1980	Not determined (no genomic data available)	Cattle	[143]
China	1988	Not determined (no genomic data available)	Mosquito (unspecified species)	[144]
Israel	2002	Genogroup IV (ISR-01)	*Culicoides imicola*	[134]
Taiwan	1992	Genogroup I (Iriki strain)	Calves	[107]
Oman	1987–1988	Genogroup I * (Unspecified strain)	Goat *Culicoides imicola*	[110]
Iran	1988–1989	Not determined	Cattle	[113]
South Africa	1968–1970	Not determined	Culicoides midges (species not specified)	[114]
Indonesia	2014	Genogroup Ib (isolate WJ-1SA/P/2014)	Cattle	[116]
Kenya	1976	Not determined	*Anopheles funestus*	[121]
Zimbabwe	1985	Not determined	*Culicoides imicola* and *C. milnei*	[115]
Turkey ^a^	2014	Genogroup II related ^b^	Sheep Goat	[128]

* Based on subsequent phylogenetic studies. ^a^ Serological and pathological detection of AKAV was performed previously. ^b^ Not included but similar to Genogroup II.

The epidemiology of arboviral diseases is deeply influenced by viral genetic evolution. Owing to the segmented RNA genome of bunyaviruses, AKAV genetic variability derives from two main sources, genetic drift and genetic reassortment. Genetic reassortment represents a major evolutionary mechanism of orthobunyaviruses that enables them to adapt and maintain viral fitness across diverse environmental conditions and host populations, thereby complicating the control and prevention of the disease. A well-known example is Ngari virus, a natural reassortant orthobunyavirus composed of the L and S segments of BUNV and the M segment of Batai virus and associated with severe febrile and hemorrhagic disease outbreaks in humans [145,146,147,148]. Experimental studies using reverse genetics have confirmed the reassortant capacity of AKAV [87]. In the field, evidence of natural reassortment events exists, including virus harboring genome constellations composed of genomic segments of different AKAV genogroups [106,136]. For instance, a reassortant virus identified in China contained an S segment derived from a Chinese strain and the L and M segments were derived from an Israeli strain [149]. LAVs may also contribute to reassortment, enhancing genetic variability of AKAV. Indeed, a Chinese AKAV isolate apparently acquired its L segment from an attenuated vaccine strain, while the M and S segments were derived from a field strain [104]. Although superinfection exclusion might occur between Simbu serogroup orthobunyaviruses [150], genetic reassortment events between viruses of the Simbu serogroup (including AKAV) have been identified, which can lead to the emergence of novel strains with altered antigenicity, virulence, tropism and/or host range [29,140,151,152], posing significant challenges for surveillance, diagnostics, and vaccine design. In the case of AKAV, limited phenotypic data are available. Recently, Na E.J. et al. (2025) showed that replacement of the S segment of AKAV-7 with that of K0505 strain, whose pathogenicities differ significantly, did not significantly reduce mortality in suckling mice, suggesting that M and/or L segments may contribute to strain-specific pathogenicity [87]. For other orthobunyaviruses such as LACV or Ngari virus, reassortment led to phenotypic consequences that altered viral replication, pathogenicity or neurovirulence [145,148,153,154], illustrating the type of phenotypic shifts that reassortment can generate within orthobunyaviruses and supporting the epidemiological and veterinary relevance of AKAV reassortment.

**Figure 5:**
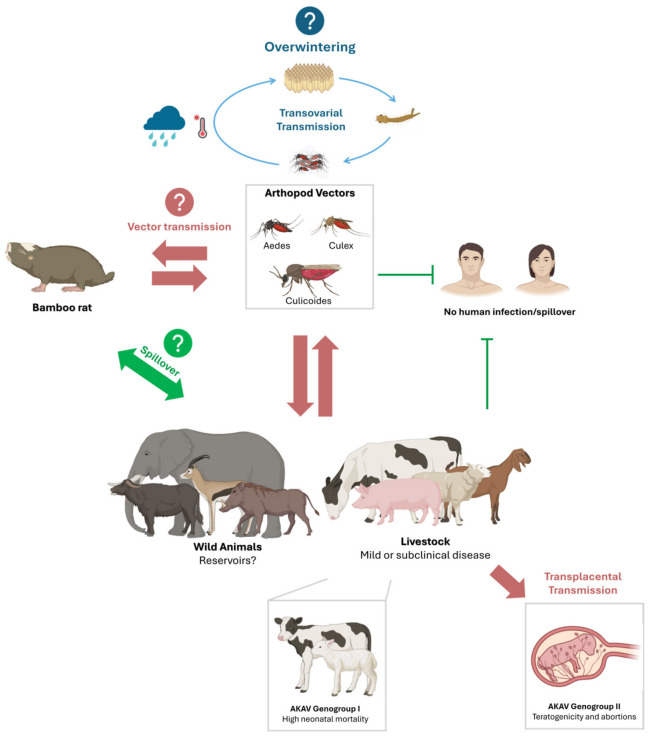
AKAV transmission cycle. AKAV can persist in an enzootic cycle involving wild or domestic ruminants and female *Culicoides* biting midges. Other non-ruminant host vertebrates can be infected by AKAV, such as elephants, warthogs or domestic pigs. To a lesser extent, mosquitoes of the genus *Aedes* or *Culex* may act as secondary vectors. No evidence of virus vertical transmission to insect vector offspring has been observed but it cannot be ruled out as a potential overwintering mechanism. AKAV infection of adult animals is usually asymptomatic or subclinical. AKAV Genogroup I infection of neonatal animal or AKAV Genogroup II transplacental transmission to fetuses lead to severe disease. Bamboo rats (and potentially other wild rodents) have been identified as susceptible AKAV hosts and may reflect a spill-over event. Insect vector transmission is the most likely infection route of bamboo, but spillovers from infected animals cannot be ruled out. Transmission to humans has not been documented.

The genetic diversity of AKAV likely characterizes the adaptability of the virus to novel susceptible host species, adding more complexity to AKAV epidemiology. Beyond cattle, a wide range of wild and domestic herbivores species have been identified as susceptible hosts [155,156] (Figure 5). Notably, different studies have demonstrated the high susceptibility of pigs to AKAV infection, with high seroprevalence reported in swine farms [157,158,159]. Experimental data suggest that domestic swine (and probably other members of the family *Siudae* such as wild boars) act as natural hosts, as they develop transient viremia that may be compatible with transmission to Culicoides vectors. Therefore, pigs may participate in the AKAV transmission cycle although direct evidence of pig-to-vector transmission is currently lacking. Experimental studies are required to determine whether swine can effectively contribute to onward AKAV transmission. Infection of pigs with AKAV leads to similar pathogenic features that resemble those observed in ruminants, including abnormal fetal development and stillbirth of piglets [158]. Considering that China sustains a high production and consumption of pork, being the world’s largest swine industry [160], the identification of swine as a highly susceptible AKAV host raises significant concerns for livestock health and productivity. Additionally, AKAV has been described as the etiological agent of three large-scale outbreaks in bamboo rat (*Rhizomys sinensis*) farms, where significant mortality rates were reported [102]. Although these findings raise questions about the epidemiological role of rodents, the marked susceptibility and severe disease observed in bamboo rats seems incompatible with a potential role in the AKAV transmission cycle, which requires some degree of disease tolerance. Instead, outbreaks in bamboo rat farms may reflect a potential spillover event, most likely vector-mediated, originated from AKAV-infected ruminants. Sporadic AKAV seroprevalence has also been detected in several non-ruminant African wildlife species [155], yet none have shown evidence of contributing to natural transmission, although they might contribute to virus maintenance. Despite the high host plasticity of AKAV that leads to broad host range, there is no epidemiological or virological evidence of human infection so far. Therefore, the zoonotic potential of AKAV is considered very low.

### 3.1. Role of Culicoides Insect Vector in Akabane Virus Spread

AKAV is a vector-borne viral pathogen primarily transmitted by adult female biting midges of the genus *Culicoides* (order Diptera, family *Cerotopogonidae*) [5,99,161,162]. Consequently, AKAV geographical distribution is constrained by the presence of competent Culicoides vector species. Several studies have identified multiple Culicoides species that are implicated in AKAV transmission, including *C. oxystoma*, *C. imicola*, *C. tainanus*, *C. punctatus*, *C. brevitarsis*, *C. schultzei complex*, *C. longipennis*, and *C. circumscriptus*, among others [99,129,131,163,164,165]. The competence of reared *C. variipennis* to AKAV has been determined after experimental oral infection, but no data are available for AKAV regarding oral infection, dissemination or transmission rates in different Culicoides species, which represents a major limitation for comparative assessments of vector efficiency. In Japan, the main Culicoides species involved in AKAV transmission is *C. oxystoma*, but *C. tainanus*, *C. punctatus*, *C. arakawae* y *C. matsuzawai* are abundantly found in cattle farms, probably playing a critical role in the AKAV transmission [163,164]. In Australia, distribution of Akabane virus depends on the seasonal distribution of *C. brevitarsis* [117,166]. In Republic of Korea, different Culicoides species such as *C. punctatus*, *C. arakawae* or *C. tainanus* are prevalent but other less abundant (*C. maculatus* or *C. oxystoma*) are also involved in AKAV transmission [167,168,169]. In China, the abundance of Culicoides species varied among cattle, sheep and goat farms, with *C. obsoletus*, *C. arakawae*, *C. tainanus*, *C. newsteadi*, *C. imicola* and *C. oxystoma* being present but their relative contribution to AKAV dissemination is unknown [170,171]. In Turkey and the Middle East, diverse Culicoides species including *C. oxystoma*, *C. imicola*, *C. schultzei*, *C. longipennis*, *C. schultzei* and *C. circumscriptus* have been identified, with some of them confirmed as AKAV vectors, which is relevant due to the presence of these species all over the Mediterranean basin [110,129,131,172]. Outbreaks of AKAV are typically seasonal and coincide with abundancy of insect vector populations, usually from late spring to autumn in temperate regions. In tropical areas with suitable conditions for Culicoides insects, AKAV circulates in annual and semi-annual cycles [173,174]. Climatic factors like rainfalls throughout the year or warmer winters can influence the biology of Culicoides vectors, facilitating the emergence of adult biting midges and increasing the risk of virus transmission [173].

Transovarial transmission has been proposed as a potential overwintering mechanism for other Culicoides-borne viruses such as SBV or EHDV [175,176]. Traditionally, AKAV has been considered incapable of vertical transmission in Culicoides vectors, but this conclusion relied on a single study published in 1990 [177]. No additional evidence regarding transovarial transmission of AKAV in Culicoides has been reported in the past three decades, which highlights a major knowledge gap. Recently, Erram D. et al. (2025) have provided experimental evidence of very low rates of vertical transmission of EHDV in *C. sonorensis* that may represent an overwintering mechanism that contributes to EHDV persistence [176]. These data overturned a long-standing assumption regarding the lack of transovarial transmission of orbiviruses. Therefore, although AKAV virus persistence and overwintering in vector populations do not seem associated with transovarial transmission in Culicoides [177], there is a need to reassess whether AKAV may also undergo transovarial transmission under specific ecological or vector-population conditions.

AKAV can replicate in cultured *Aedes albopictus* cells like other Culicoides-transmitted viral diseases [178,179,180]. In Japan, AKAV has been isolated from *Aedes vexans nipponii*, *Culex tritaeniorhynchus*, *Aedes sinensis*, *Anopheles vagus* and *Culex quinquefasciatus* in nature [8,103,105,181], although isolation does not reflect vector competence. Despite the fact that alternative arthropod vectors to Culicoides midges might not significantly contribute to AKAV transmission [182], the potential role of other hematophagous insects should be clarified as it could imply additional challenges for the prevention and control of this arthropod-borne virus. Indeed, if mosquitoes of the genus *Anopheles*, *Culex* or *Aedes* are identified as competent for AKAV, transmission by these alternative vectors could broaden the host range of AKAV and potentially facilitate its persistence between periods of low Culicoides activity by keeping the virus circulating in nature or allowing for virus transovarial transmission.

Epizootic events involving AKAV are typically seasonal and depend on the abundancy of insect vector populations [173,174]. Nonetheless, human and environmental factors influence the dynamics of vector-borne diseases by altering the biology, ecology, and distribution of arthropod vectors [183]. In recent decades, the global arbovirus distribution has expanded because of multiple anthropogenic factors, including international livestock movement, changes in agricultural practices, urbanization, and global warming [184,185,186]. Among them, global warming is a major driver that enhances the extrinsic incubation period, vector survival, reproduction rates, and the geographic distribution of competent insect vectors [187]. Expansion of AKAV to northern latitudes may occur because of substantial expansion of a suitable habitat for Culicoides vectors [188]. Indeed, as an example, the Culicoides-transmitted Bluetongue virus and Epizootic hemorrhagic disease virus have expanded their distribution in recent decades to colder northern regions [189,190]. Passive long-distance dispersion of infected Culicoides biting midges from affected areas contributes to distribution of these orbiviral diseases within affected regions and facilitates virus introduction to non-endemic regions, as recently observed in Europe [1,191,192,193]. Wind-mediated transport of infected Culicoides could also be a driver of AKAV virus emergence in non-affected areas. Although AKAV circulates in the Mediterranean basin, recognized as a hotspot for arbovirus transmission between territories, European countries have not detected AKAV circulation within their territories yet. Considering that Culicoides species involved in AKAV transmission include those also present in Europe, the spread of AKAV to non-endemic European territories with naïve livestock populations is quite feasible and should be a matter of concern.

Alternative transmission routes may exist for AKAV. Although vertical transmission in mammals does not contribute to AKAV spread among adult animals, it is characteristic of AKAV. As stated before, AKAV significantly affects fetuses as it can efficiently cross the placental barrier, leading to fetus infection during critical developmental stages. Transplacental transmission of AKAV has been experimentally demonstrated in laboratory animal models such as pregnant hamsters, where the virus showed the ability to establish a transplacental infection, replicating at high level in placental and fetal tissues, particularly the brain [194]. Experimental infection of pregnant sheep and cattle resulted in mild disease and viremia in adult animals, while fetuses developed systemic infection and a marked viral tropism in the brain, leading to developmental abnormalities [195,196,197]. AKAV vertical transmission has been also demonstrated in pregnant goats [198]. In high-density farms, alternative horizontal transmission routes might contribute to AKAV spread. Epizootic events in bamboo rat farms were characterized by high prevalence of AKAV disease [102]. Although insect vectors are likely the main source of virus transmission, this homogeneous spread among bamboo rats might indicate potential direct contact or fecal-oral transmission, especially if we consider the highly sociable and coprophagous behavior of rodents. Indeed, experimental oronasal infection of pigs led to productive AKAV replication [159], demonstrating that swine can also acquire the virus through direct mucosal exposure although this route appears to require high viral titers to establish infection.

### 3.2. Diagnosis and Surveillance

Surveillance of AKAV is particularly challenging because infected ruminants develop short-lived viremia and often remain asymptomatic or exhibit only mild clinical signs [199], complicating diagnosis. Direct AKAV detection can be achieved through virus isolation from blood samples collected during the viremic phase and, when available, from fetal tissues [200]. Mammalian cell lines such as Vero, BHK-21 and HmLu-1, as well as mosquito-derived cell lines including C6/36 or KC Culicoides cells, are used for virus isolation. Viral identity can subsequently be confirmed by immunofluorescence or immunohistochemistry assay using monospecific or monoclonal antibodies [201,202]. However, virus isolation is time-consuming and highly dependent on sample quality and the timing of sample collection, which limits its applicability in routine surveillance programs [200]. Most AKAV-specific molecular assays target conserved regions of the S genome segment encoding the N protein, as this genomic region exhibits a high degree of conservation among AKAV isolates. First, RT-PCR (Reverse Transcription Polymerase Chain reaction) assays were primarily used for molecular characterization and genetic comparison of AKAV isolates from different geographical regions [203]. Subsequently, a nested RT-PCR assay enabled the specific detection and differentiation of AKAV and AINOV through virus-specific primers targeting the S segment [204]. Later, a multiplex real-time RT-PCR assay allowed for the simultaneous detection and quantification of AKAV and AINOV in a single reaction [205], while one-step multiplex RT-qPCR assays enabled the differential detection of AKAV, SBV and AINOV [206]. More recently, RT-LAMP (Loop-Mediated Isothermal Amplification) assays targeting conserved regions of the N gene have been proposed as rapid alternatives for AKAV RNA detection [207].

Although the molecular diagnostic approaches provide sensitive and specific detection during the acute phase of infection, their diagnostic value remains limited by the transient nature of AKAV infection. Therefore, AKAV surveillance mostly relies on serology. Commonly used serological approaches include neutralization tests (NTs) (Serum NTs (SNTs) or Virus NTs (VNTs) [200]), immunofluorescence assays and enzyme-linked immunosorbent assays (ELISAs). SNT and VNT are generally considered the gold standard for AKAV diagnosis because of their high specificity and their ability to detect functional neutralizing antibodies. However, they are time-consuming and require live virus and cell culture, being less suitable for surveillance programs. ELISAs are better adapted for high-throughput screening, and several competitive commercial kits are currently available for AKAV serology [208,209]. Some ELISA assays are based on the N protein [210,211], which is a highly immunogenic antigen. However, N-based ELISAs are conditioned by serological cross-reactivity due to high conservation of the N protein among the Simbu serogroup [212], although N-specific monoclonal antibodies can improve the specificity of AKAV detection in a double antibody sandwich ELISA [210]. Alternatively, ELISA based on whole inactivated virus can solve cross-reactivity [209,213]. Recently, serological assays based on the viral glycoprotein Gc have been developed. Gc protein contains the major neutralizing epitopes and exhibits greater antigenic variability than the N protein. Hence, Gc-based triplex ELISAs have been developed, improving the differentiation of seroconversion against SBV, AKAV and Shuni virus (SHUV) [214]. Studies using recombinant truncated Gc proteins identified neutralizing domains located within amino acids 1–97 and 189–397, which may support the use of defined Gc regions as diagnostic antigens [41]. More recently, a highly conserved neutralizing epitope located in the C-terminal region of AKAV Gc (1134-SVQSFDGKL-1142) was identified using neutralizing monoclonal antibodies [42]. This epitope was subsequently incorporated into chimeric virus-like particles (VLPs) based on the hepatitis B virus core antigen (HBcAg), being subsequently used as an antigen in a preliminary indirect ELISA for AKAV antibody detection [215]. Although these Gc-based and epitope-specific approaches are promising, most require robust evaluation before a potential implementation in surveillance programs. Moreover, surveillance in vaccinated populations remains problematic because validated DIVA (Differentiating Infected from Vaccinated Animals) systems are not currently available for AKAV. Consequently, distinguishing antibodies induced by natural infection from those elicited by vaccination remains a major limitation for epidemiological surveillance and disease control programs, particularly due to the use of classical vaccine approaches.

## 4. AKAV Disease

As previously stated, AKAV was identified as the etiological agent of congenital malformations in cattle during Japanese epizootics of last century [7,8,91]. Depending on the timing of infection and the age of the animal, AKAV infection results in diverse clinical manifestations. Adult cattle infected with AKAV typically develop transient viremia without evident clinical signs [216,217], although AKAV infection may lead to encephalomyelitis and neurologic signs in some cases [218,219,220]. Postnatal infection of calves with certain AKAV strains, particularly the Iriki strain and related Genogroup I isolates, can cause severe neurological disease characterized by encephalomyelitis and neurological symptoms such as astasia, ataxia, opisthotonus, hypersensitivity and behavioral abnormalities [98,141,221]. Transplacental transmission is a hallmark of AKAV infection. AKAV crosses the placental barrier during pregnancy, leading to fetal infection and congenital malformations whose severity varies depending on the gestational stage at which fetal infection occurs [119]. Usually, fetuses infected with AKAV between three and eight months of gestation develop congenital defects although the highest incidence of congenital abnormalities occurs when fetal infection takes place between the third and sixth months of pregnancy [119]. The major congenital abnormalities observed in affected fetuses include hydranencephaly, porencephaly, arthrogryposis, microencephaly, and cerebellar hypoplasia [119,222,223]. Affected calves may be stillborn, born prematurely, or delivered at term with severe deformities that are fatal within days of birth [222,223].

AKAV shows marked neurotropism, being isolated from the brain, spinal cord and cerebrospinal fluid of infected fetuses, although systemic fetal infection is frequently observed [219,224,225]. The virus targets neuroglial cells in the brain stem and neuronal cells, particularly in the ventral horn of the spinal cord where neuronal cell loss occurs [202,221]. The main lesion observed in infected fetuses is non-purulent encephalomyelitis affecting the undifferentiated central nervous system, accompanied by necrosis of neural tissue [222,223]. AKAV infection of fetuses also affects skeletal muscle during the myotubule phase of development, leading to severe muscle atrophy [222,224].

In sheep and goats, AKAV infection leads to a clinical outcome that resembles that observed in cattle, being characterized by either postnatal disease or congenital disease depending on the characteristics of the viral strain [118,195,198,220,226,227]. Neurological abnormalities in affected small ruminants include arthrogryposis, hydranencephaly, kyphosis, scoliosis, brachygnathia, microencephaly, and porencephaly [195,198,226]. In infected lamb fetuses, AKAV preferentially infects neuronal, neuroglial and ganglionic cells, inducing pathological features such as atrophy of skeletal muscles and non-purulent encephalomyelitis affecting different brain regions [195,226,228].

As previously stated, AKAV can infect non-herbivores species such as pigs or bamboo rats. Although adult pigs are usually asymptomatic, swine are susceptible hosts in nature and may serve as silent hosts in the virus-host-vector AKAV circulating cycle [157,158,159,229]. Transplacental infection of pig fetuses results in a severe congenital disease similar to that observed in ruminant hosts, which indicates that AKAV virulence and pathogenicity are comparable across species. In this regard, AKAV (Genogroup I isolate) has been isolated from stillborn and aborted porcine fetuses, being identified as the causative agent of congenital malformations including hydranencephaly, arthrogryposis, spinal curvature and skeletal muscle atrophy [92,158]. Similar to affected calves, infected piglets also display nonsuppurative encephalomyelitis and decreased neuronal density in the ventral horn of the spinal cord. Indeed, AKAV infection can be detected in neuronal and glial cells of the brain stem and spinal cord of piglets [92,158].

The known host range of AKAV was further expanded after identification of natural AKAV infection in bamboo rats. Notably, high morbidity and mortality rates were reported among adult bamboo rat populations [102], which differ from the usual subclinical or asymptomatic disease in adult livestock. Affected animals displayed typical nonsuppurative encephalomyelitis in central nervous tissues accompanied by meningeal and perivascular infiltration by mononuclear cells, but other organs such as lung, heart, kidney, liver or spleen were also targeted, showing gross and severe histopathological lesions. The systemic infection and marked virulence in adult bamboo rats suggest distinct infection dynamics and pathogenesis compared to ruminant hosts.

Different laboratory animal species, including hamsters, rats, guinea pigs or mice, have been used as experimental animal models for AKAV research. Following AKAV inoculation, hamsters, suckling rats and guinea pigs exhibit systemic infection and replication in the central nervous system, displaying pathological features observed in natural AKAV host, like nonsuppurative encephalomyelitis and skeletal muscle atrophy. Therefore, these species have been used for studying AKAV vertical transmission, fetal infection, neurovirulence or viral pathogenicity and tropism [194,230,231,232]. Suckling mice have been widely used to study strain virulence, virulence factors, neuroinvasiveness, and organ tropism, as they consistently develop severe neurological disease after infection [14,62,87,89,217,233,234,235,236], but the immature immune system limits their application in vaccine studies. Immunocompetent adult mice have been used for AKAV research, but they are less susceptible to AKAV infection than newborn mice and usually require intracerebral inoculation to establish infection in the nervous system [233,234], which limits to some extent their applicability. Alternatively, type I interferon receptor knock out (IFNAR(−/−)) mice, widely employed for arbovirus research [237,238,239,240], have been recently characterized as a mouse model for AKAV study in vivo [241]. IFNAR(−/−) mice show high susceptibility to intraperitoneal or subcutaneous inoculation, although no neuropathogenesis has been reported after infection, which may constrain implementation of this mouse model for evaluating AKAV pathogenicity and neurovirulence. In any case, IFNAR(−/−) mice constitute a highly permissive system for studying AKAV replication and a homogenous animal model for AKAV vaccine evaluation [241], an aspect that is unattainable in immature newborn mice. However, the impaired type I interferon signaling represents an inherent bias that may overestimate vaccine protection and does not fully reflect the antiviral mechanisms of immunocompetent ruminants.

## 5. Vaccine Approaches Against Akabane Virus

Due to the substantial economic impact of AKAV on the primary sector and the absence of effective therapeutic treatments against the virus, vaccination remains as the only measure with proven effectiveness to control this viral disease [242]. The first vaccine approaches against AKAV were based on conventional live-attenuated and inactivated vaccines. These are currently commercialized in different countries where AKAV is endemic, particularly in Australia, Japan and Republic of Korea [242]. A LAV was first developed by Kurogi et al. (1978) through attenuation of the highly virulent OBE-1 strain by extensive passage in cell culture [243]. After experimental inoculation of mice, calves and pregnant cows, the attenuated AKAV strain TS-C2 showed reduced virulence while maintaining a high immunogenic profile, inducing nAbs against the virus [244]. Indeed, the attenuated strain TS-C2 induced robust protection against challenge with virulent OBE-1 strain in immunized pregnant cows [245,246]. Subsequently, other attenuated strains of AKAV showed similar results in terms of immunogenicity and protection in pregnant animals [247]. LAVs offer advantages related to the induction of strong humoral and cellular immune responses, which correlate with robust protection compared to inactivated vaccines. However, several factors should be considered, including DIVA incompatibility, possible reversion to virulence and teratogenicity, possible transmission to insect vectors or the risk of genetic reassortment between vaccine and field strains that may contribute to viral diversity as recently suggested [87,104]. As a safer but less immunogenic alternative [248], inactivated vaccines against AKAV were developed. Formalin-inactivated virus demonstrated high protective efficacy in calves and pregnant cows against virulent challenge with AKAV, preventing viremia and fetal infection [243]. In the field, immunization of pregnant cattle with adjuvanted formalin-inactivated AKAV induced high seroconversion rates characterized by the induction of nAbs against the virus while maintaining an adequate biosafety profile [243]. Lately, novel inactivated vaccines against AKAV are being developed [249,250]. However, inactivated vaccines also lack DIVA compatibility, since crude extract of produced viruses also contains NSs and NSm antigens.

As defined by Lauer, Borrow and Blanchard (2017), multipathogen/multidisease vaccines target protective antigens from two or more pathogens to confer immunity against multiple diseases [251]. Designing vaccine strategies that target multiple diseases can provide a reduction in time and costs of production, allowing for faster and more effective vaccination campaigns. Recent multipathogen approaches using inactivated AKAV aimed to develop bivalent or trivalent vaccines against AKAV and other ruminant viruses (Table 2). Following a massive outbreak of AKAV and bovine ephemeral fever virus (BEFV) in Republic of Korea [252], a bivalent inactivated vaccine candidate targeting both AKAV and BEFV was assessed, showing promising results in terms of immunogenicity in laboratory animal models and natural hosts of both diseases such as sows and cattle [253]. Similarly, Kim et al. (2011) developed a safe and efficacious inactivated trivalent vaccine against AKAV, AINOV, and Chuzan virus (CHUV), as these viruses potentially co-circulate in East Asia [254] (Table 2). Similar to AKAV, both AINOV (genus *Orthobunyavirus*, family *Bunyaviridae*) and Chuzan virus (genus *Orbivirus*, family *Sedoreoviridae*) are associated with reproductive and congenital disorders in ruminants [255,256,257,258]. The inactivated trivalent vaccine (Nisseiken Bovine Abnormal Parturition Trivalent Inactivated Vaccine, Nisseiken Co., Ltd., Tokyo, Japan) was shown to be safe in mice, guinea pigs, and pregnant cows. After field application, immunized animals developed strong nAb responses against all three viral pathogens. Although the inactivated trivalent vaccine demonstrated protective efficacy against each virus, these results were conditioned by the likely attenuation of the inocula used in the challenge experiments [254].

The development of RG systems for AKAV provides the possibility to generate novel biotechnological tools to dissect virus biology and pathogenesis, and also enables the design of genetically manipulated LAVs with improved safety profiles [86,88,89,90,259,260]. In this sense, mutations in the L and M segments of AKAV are related by attenuated phenotypes in suckling mice [14]. AKAV deletion mutants lacking the NSs gene are also associated with significant attenuation in suckling mice and pregnant goats while maintaining adequate humoral immune responses, indicating that knockout of this virulence factor could support the rational engineering of theoretical DIVA-compatible LAVs against AKAV [85,86,87,260], as observed for other bunyaviruses such as RVFV or SFTSV [78,79]. Interestingly, RVFV mutants lacking NSs expression were proposed as safe viral vector platforms capable of expressing antigens of different ruminant diseases [261,262,263]. A similar strategy could also be explored to design multipathogen vaccines based on genetically attenuated AKAV.

The protective efficacy of LAVs and inactivated vaccines mainly relies on the presentation of the immunodominant Gc glycoprotein, which contains major neutralizing epitopes. The Gc protein is a key antigen in vaccine development against bunyaviruses due to its prominent role in virus entry [38]. Apart from multipathogen inactivated vaccines, an innovative bivalent approach against AKAV and SBV was developed through the design of a concatemer composed of two N-terminal domains (N-terminal 234 residues) of the Gc glycoproteins of SBV and AKAV [264] (Table 2). These regions predominantly harbor the neutralizing epitopes found within the Gc proteins of SBV and AKAV [39,41]. The construct conferred robust protection against SBV challenge in immunized IFNAR(−/−) mice and cattle, effectively impairing viral replication [264]. Although its protective efficacy against AKAV was not tested, this recombinant vaccine candidate induced a neutralizing humoral response against AKAV, which may act as a correlate of protection as suggested in previous studies [254,265]. Lately, Ogawa, Y. et al. (2022) [41] identified two N-terminal regions of the AKAV Gc protein (aa 1–97 and 189–397) as targets of nAbs (Table 2). The neutralizing responses elicited in mice immunized with these regions, either individually or fused, support their potential as subunit vaccine candidates against AKAV [41]. Nonetheless, the N-terminal portion of the Gc protein, especially the head domain (main target of nAbs), is likely subjected to strong immunological pressure and can be considered a hypervariable region among AKAV strains, which could contribute to immune scape or asymmetric cross-protection between genogroups and isolates [40,217,242,266]. Alternatively, Wang, J. et al. (2024) [42] identified a neutralizing epitope within the C-terminal region of AKAV Gc protein that is highly conserved among AKAV genogroups. Importantly, coating of hepatitis B virus core antigen virus like-particles with the identified epitope led to AKAV-neutralizing responses in immunized BALB/c mice [42,215]. Nonetheless, a monoclonal nAb directed against this conserved neutralizing epitope protected mice from lethal challenge with AKAV, representing a novel therapeutic alternative against AKAV [267]. Overall, these works provide important insights for the rational design of DIVA vaccines against AKAV and related orthobunyaviruses and highlight the complexity of Gc-based antigen design and the requirements related to proper protein folding and posttranslational modifications (particularly glycosylations) for successful Gc-based AKAV vaccine development.

Cross-reactive immune responses, including cross-neutralizing in some cases, are frequently observed among viruses of the Simbu serogroup [212,268]. Considering this cross-reactivity, the efficacy of the AKAV, AINOV and CHUV inactivated trivalent vaccines was evaluated against SBV, closely related to AKAV and AINOV. Nonetheless, this multipathogen vaccine failed to induce any degree of protection against SBV in immunized cattle [269]. As discussed by the authors, cross-neutralizing responses within the Simbu serogroup may not be bidirectional [269]. Nonetheless, although the trivalent vaccine did not elicit cross-neutralizing antibodies against SBV, a humoral immune response against the N protein of SBV was observed before SBV challenge [269]. This observation is relevant as the M segment of AKAV, which encodes the viral envelope glycoproteins Gn and Gc and the NSm protein, exhibits variability among AKAV genogroups and isolates [136], and neutralizing epitopes within the Gc glycoprotein sequence can differ between them, illustrating the evolution of AKAV protein Gc due to immunological pressure [270]. Therefore, the evaluation of alternative antigens that are conserved among AKAV isolates and across viruses of the Simbu serogroup could be of interest. N-specific antibody cross-reactivity has been observed among viruses of the Simbu serogroup [271,272] and, for SBV, the N protein constitutes a source of CD8+ T cell epitopes and contributes to protective immunity when formulated as a vaccine antigen [273,274]. Recently, Soares-Guerra, G. et al. (2023) proposed the N-terminal domain (aa 1–133) and a smaller fragment designated C4 (aa 1–58) of the SBV N protein as broadly protective antigens against viruses of the Simbu serogroup owing to their high degree of conservation (over 85% sequence homology with homologous regions of AKAV N protein) [69]. Although both N-truncated vaccine candidates protected immunized IFNAR(−/−) mice against challenge with SBV, their capacity to protect against other orthobunyaviruses, including AKAV, remains entirely untested. For this reason, N-based approaches should be considered as a preliminary and unvalidated concept, pending experimental evidence of cross-protection across orthobunyaviruses. Nevertheless, the identification of CD8+ T cell epitopes within the SBV N protein and the protective effect observed for N-based vaccine candidates [69] suggest that cellular immune responses contribute to protection through mechanisms that are not exclusively dependent on neutralizing antibodies. However, cell-mediated immunity against AKAV remains poorly characterized. To date, only limited evidence is available regarding AKAV-specific T cell responses. Wang et al. (2025) reported increases in CD4+ and CD8+ T cell populations after vaccination with formaldehyde-inactivated AKAV vaccine [250], suggesting that cell-mediated immunity may contribute to vaccine-induced protection along with antibody-mediated immunity. In any case, further research is needed to better characterize AKAV-specific cellular immune responses. Studies conducted with other orthobunyaviruses provide useful insights into the potential relevance of T cell-mediated immunity. Robust CD4+ and CD8+ T cell responses against LACV were identified in mice. These polyfunctional responses were characterized by the production of IFN-γ, TNF-α, IL-2 and granzyme B. Importantly, Gc and the N antigens were identified as immunodominant targets of cytotoxic T cell responses. Experimental vaccine candidates designed to enhance T cell responses against Gc or N protein improved cytotoxic T cell activity and protection [275]. Indeed, resistance to LACV infection is associated with stronger IFN-γ-producing CD8+ T cell responses in adult mice [276]. For OROV, lipid nanoparticles containing glycoproteins from different OROV lineages induced potent CD4+ and CD8+ T cell responses along with a strong humoral immunity and cross-neutralizing antibodies [277]. Similarly, a VLP-based mRNA vaccine against OROV generated through the co-expression of the M polyprotein and N protein induced both humoral and cellular immune responses, with a predominant Th1 biased profile. Furthermore, the authors identified a highly conserved putative immunodominant epitope within the N protein [278]. Overall, these findings indicate that antigen-specific cellular immune responses contribute to protection against orthobunyaviruses. Therefore, future AKAV vaccine studies should aim for a more comprehensive characterization of both humoral and cellular immune responses, with particular attention to T-cell-mediated immunity and the contribution of conserved antigens such as N to protective responses.

The development of effective vaccines is crucial for maintaining herd immunity and reducing viral circulation in endemic areas. Maternal immunity represents a potential factor that may condition AKAV vaccination strategies. During pregnancy, maternal AKAV-nAbs cross the placental barrier [91]. After birth, maternal antibodies transfer occurs primarily through colostrum consumption. Although maternally derived antibodies provide early protection, interference with vaccine effectiveness may occur, particularly in the case of LAVs [279]. Therefore, understanding the duration of maternal antibody persistence is critical for determining optimal vaccination timing in young animals. In calves, the estimated duration of AKAV maternally derived antibodies ranges from 4 to 5 months after birth, although it may vary depending on factors such as vaccination of dams, cattle type or geographic location [280,281]. Further research should be performed to clarify the impact of maternal immunity on AKAV vaccine implementation across different ruminant species and its implications for herd immunity and viral circulation.

Although no therapeutic treatments are currently available against AKAV, protoporphyrin IX, a heterocyclic organic compound precursor of heme [282], has been identified through structure-based molecular docking as a potent virucidal agent against this virus [283]. This molecule targets the AKAV glycoprotein, impairing virus entry in vitro and in vivo through a potential blocking of the fusogenic activity of the Gc glycoprotein [283]. Although these results are promising, vaccination remains as the most effective measure for AKAV control.

## 6. Conclusions

AKAV is a highly adaptable arbovirus whose epidemiology is constantly evolving due to climate-driven vector expansion, increasing ecological opportunities, and rapid genetic evolution. Its marked host plasticity, being able to infect a broad spectrum of mammalian hosts such as cattle, small ruminants, pigs, and even rodents, amplifies transmission networks and hampers prediction of outbreak dynamics. Combined with the expanding distribution of competent *Culicoides* species, changes in AKAV geographic range and pathogenic profile will likely occur as environmental pressures intensify. This evolving scenario underscores the need for forward-looking strategies that should prioritize the characterization of AKAV determinants of virulence, pathogenicity, replication, transmission, host–virus interaction and susceptibility, and the evaluation of vector competence; surveillance of regions undergoing climatic transitions where Culicoides vectors are expanding, including AKAV-free countries; monitoring of key Culicoides species such as *C. brevitarsis*, *C. imicola* and the regionally dominant Culicoides species that preferentially transmit Simbu serogroup viruses; systematic sequencing of circulating AKAV strains to identify genetic features associated with higher pathogenic or transmission potential; and development of next-generation vaccines that cover representative genotypes and incorporate antigenically relevant and protective conserved regions based on immunogenicity and protection studies of AKAV antigens. Development of next-generation vaccines based on subunit, DNA, RNA or viral vector platforms, as well as genetically engineered platforms may also enable future serologic-DIVA compatibility, a critical requirement for reliable surveillance, outbreak investigation, AKAV-free status certification and international trade. Considering the uncertainty of a potential future expansion into currently AKAV-free regions, strengthening these priorities will be essential to mitigate the growing economic impact of this evolving livestock pathogen.

**Table 2:** Vaccine approaches against AKAV.

Vaccine Type	Strain/Antigen	Animal Model	Dose	Inoculation Route	Adjuvant	Immunogenicity	DIVA	Challenge	Protection	Ref.
Engineered LAV	AKAV∆NSs (TS-C2 strain)	Pregnant goats	2.3–4.6 × 10^7^ PFU	I.V.	No	nAb response against AKAV	Potentially DIVA	Not challenged	-	[85,260]
Multipathogen Inactivated Vaccine	AKAV (KV0505 strain) and BEFV (DS11 strain)	Mice (no strain specified)	Two doses (not specified, BEI inactivated)	I.P.	Montanide IMS1313VG/Montanide IMS1313VG+ revibFlaB protein	nAb response against AKAV and BEFV	No	Not challenged	-	[253]
				I.M.						
		Guinea pigs		I.M.						
		Sows								
		Cattle								
	AKAV (OBE-1 strain), AINOV (JaNAr28 strain) and CHUV (K-47 strain)	Adult female ICR mice	3 × 10^7.3^ TCID_50_ (AKAV), 3 × 10^7.5^ TCID_50_ (AINOV), 3 × 10^6.8^ TCID_50_ (CHUV) (two doses, BEI inactivated)	S.C.	Montanide^®^ IMS 1314	nAb response against AKAV, AINOV and CHUV. No differences between formalin and BEI inactivation.	No	Not challenged. Safety test. No adverse effects after immunization.	-	[254]
			3 × 10^7.3^ TCID_50_ (AKAV), 3 × 10^7.5^ TCID_50_ (AINOV), 3 × 10^6.8^ TCID_50_ (CHUV) (two doses, formalin inactivated)							
		Adult Hartley female guinea pigs	3 × 10^7.3^ TCID_50_ (AKAV), 3 × 10^7.5^ TCID_50_ (AINOV), 3 × 10^6.8^ TCID_50_ (CHUV) (two doses, BEI inactivated)	I.P.						
			3 × 10^7.3^ TCID_50_ (AKAV), 3 × 10^7.5^ TCID_50_ (AINOV), 3 × 10^6.8^ TCID_50_ (CHUV) (two doses, formalin inactivated)							
		3–7 months old pregnant Holstein–Friesian cows	3 × 10^7.3^ TCID_50_ (AKAV), 3 × 10^7.5^ TCID_50_ (AINOV), 3 × 10^6.8^ TCID_50_ (CHUV) (two doses, BEI inactivated)	I.M.						
			3 × 10^7.3^ TCID_50_ (AKAV), 3 × 10^7.5^ TCID_50_ (AINOV), 3 × 10^6.8^ TCID_50_ (CHUV) (two doses, formalin inactivated)							
		Pregnant and non-pregnant Holstein–Friesian cows	3 × 10^7.3^ TCID_50_ (AKAV), 3 × 10^7.5^ TCID_50_ (AINOV), 3 × 10^6.8^ TCID_50_ (CHUV) (two doses, formalin inactivated)	I.M.		Field trial. nAb response against AKAV, AINOV and CHUV.		Not challenged	-	
		3–5 months old pregnant Holstein–Friesian cows	3 × 10^7.3^ TCID_50_ (AKAV), 3 × 10^7.5^ TCID_50_ (AINOV), 3 × 10^6.8^ TCID_50_ (CHUV) (two doses, formalin inactivated)	I.M.		nAb response against AKAV, AINOV and CHUV.		5 × 10^7^ TCID_50_ of each AKAV, AINOV and CHUV	Protection observed in immunized animals ^a,c,^*	
		9.4 months old female Holstein–Friesian calves	3 × 10^7.3^ TCID_50_ (AKAV), 3 × 10^7.5^ TCID_50_ (AINOV), 3 × 10^6.8^ TCID_50_ (CHUV) (two doses, formalin inactivated)	I.M.	Aluminum (III) chloride hexahydrate	nAb response against AKAV, AINOV and CHUV. Humoral response against the SBV protein N.		SBV passaged in cattle.	No protective efficacy against SBV	[269]
Subunit Vaccine	^1^ N-terminal 234 aa of Gc protein of AKAV	IFNAR(−/−) Mouse	20 μg (two doses)	S.C.	GERBU adjuvant Mm	Humoral immune response against constructs	Yes	SBV (strain BH619/12). Not challenged with AKAV.	No protective efficacy against SBV	[264]
	^1^ Covalently linked N-terminal 234 aa of Gc protein of SBV and AKAV	IFNAR(−/−) Mouse							Complete protection against SBV ^b,c^	
		Cattle	50 μg (two doses)		POLYGEN™ adjuvant	nAbs against SBV and AKAV		SBV passaged in cattle. Not challenged with AKAV.	Complete protection against SBV ^c^	
	^2^ Truncated Gc protein (aa 1−97) (OBE-1 strain)	5 weeks old female BALB/c mice	100 μg (two doses)	S.C.	Freund’s complete adjuvant/Freund’s incomplete adjuvant		Yes			
	^2^ Truncated Gc protein (aa 189−387) (OBE-1 strain)					nAbs against AKAV (OBE-1 strain)		Not challenged	-	[41]
	^2^ Truncated Gc protein (aa 1−97 fused to aa 189−387) (OBE-1 strain)									
	^3^ Truncated Gc protein (aa 991−1232) (TJ2016 isolate)	6–8 weeks old female BALB/c mice	100 μg (four doses)	S.C.	Freund’s complete adjuvant/Freund’s incomplete adjuvant	nAbs response against AKAV based on mAb production (TJ2016 isolate)	Yes	Not challenged	-	[42,215]
	^2^ Gc neutralizing epitope (aa 1134−1142)	Not evaluated in animal model	-	-	-	-	Yes	-	-	
	VLPs coated with Gc neutralizing epitope (aa 1134−1142)	6 weeks old female BALB/c mice	5 μg	I.M.		nAbs against AKAV (TJ2016 strain)	Yes	Not challenged	-	
			25 μg							

Expressed by ^1^ mammalian, ^2^ *E. coli* or ^3^ baculovirus expression system; ^a^ absence of clinical signs; ^b^ absence of weight loss; ^c^ absence of viremia, RNAemia and RNA in target organs; * Results conditioned by potentially attenuated challenge viruses. Inoculation: IM, intramuscular; SC, subcutaneous; IP, intraperitoneal. “Potentially” indicates theoretical DIVA compatibility pending experimental validation.

## Data Availability

The original contributions presented in this study are included in the article. Further inquiries can be directed to the corresponding author.
